# Impact of the “Amendments to the Act of the Protection of Personal Information” to Global Health Research Conducted in Japanese Medical Facilities

**DOI:** 10.2188/jea.JE20220141

**Published:** 2022-09-05

**Authors:** Soichiro Saeki

**Affiliations:** Center Hospital of the National Center for Global Health and Medicine, Tokyo, Japan

On April 1, 2022, the amended Act of the Protection of Personal Information in Japan (APPI) began to be enforced. This amendment has made the previous privacy law in Japan stricter, disabling the use of personal information outside of the intended and agreed use among providers.

Although such restrictions may seem natural, the impact this has on research being conducted in medical facilities in Japan is enormous. As this amendment prohibits the use of personal information outside its intended use, research using patient data obtained in clinical settings may become restricted. More precisely, unless the medical institution has a policy of conducting research as well as clinical practice, such as university hospitals and national research and development agencies, written consent must be obtained from all patients recruited for research projects to agree to the precise content of the specific research project. In other words, there is a possibility that future projects conducted retrospectively under previously obtained clinical data may be restricted in most medical facilities, even when researchers obtain approval of the ethical committee to gain opt-out consent from the patients.

This amendment could hinder global health research conducted in Japan. Recent studies regarding foreign patients published in the *Journal of Epidemiology* in Japanese medical facilities have been conducted using retrospective review of healthcare records.^1^^,^^2^ As foreign patients in Japanese medical facilities are still a minority,^3^ the gold standard for research in a sample that accounts only for a small portion of the total cases would be a retrospective, case-control study.^4^ If such studies were to be conducted in the future, written consent from literally all patients would be required. Especially for foreign patients, consent must be obtained simultaneously, as there is no guarantee that contact would be possible again. Foreign patients may reside in far-off locations, possibly outside the country. Linguistic barriers would make the process of gaining consent via mail or online more cumbersome than among native patients. Researchers must overcome such high barriers in order to conduct even a single-center retrospective study. Such obstacles would limit research conducted in many medical facilities. However, data regarding foreign patients in Japan are still lacking.^2^^,^^3^ Now is the time to promote such research, rather than limiting it.

Furthermore, this limitation applies to other studies that have been, and are to be conducted in Japan. Under the amended APPI, retrospective reviews of healthcare records under the name of research would be strictly limited in many hospitals. This may create a strong selection bias among studies in Japan.

However, as there is currently no guidance of the amendment of the APPI from the relevant governmental agencies, although the legislation has already begun to be enforced, to what extent such restrictions apply are still unclear. After the announcement from the Ministry of Health, Labour and Welfare,^5^ some academic organizations are now acquiring information to determine the impact of the amendment.^6^ Although fully acknowledging the importance of respecting the privacy and protecting personal information of patients, it is hoped that some compromise can be reached to determine an optimum solution to promote research while fully protecting the rights of patients in Japan.
